# Dual Bronchodilators in Bronchiectasis Study: a randomised controlled trial

**DOI:** 10.1183/23120541.01079-2024

**Published:** 2025-06-02

**Authors:** Nina Wilson, Miranda Morton, Tara Homer, Ann Breeze Konkoth, Richard Joyce, Anneka Kershaw, Hazel Wilde, Alison Liddle, James Wason, Laura Ternent, Maria Allen, Robert Lord, John Steer, Graham Devereux, James D. Chalmers, Adam T. Hill, Charles S. Haworth, John R. Hurst, Anthony De Soyza

**Affiliations:** 1 Biostatistics Research Group, Population Health Sciences Institute, Newcastle University, Newcastle upon Tyne, UK; 2 Newcastle Clinical Trials Unit, Population Health Sciences Institute, Newcastle University, Newcastle upon Tyne, UK; 3 Health Economics Group, Population Health Sciences Institute, Newcastle University, Newcastle upon Tyne, UK; 4 The Newcastle upon Tyne Hospitals NHS Foundation Trust, Newcastle upon Tyne, UK; 5 Wythenshawe Hospital Manchester University NHS Foundation Trust, Manchester, UK; 6 North Tyneside General Hospital, Northumbria Healthcare NHS Foundation Trust, North Tyneside, UK; 7 Department of Clinical Sciences, Liverpool School of Tropical Medicine, Liverpool, UK; 8 Molecular and Clinical Medicine, School of Medicine, University of Dundee, Dundee, UK; 9 Centre for Inflammation Research, Institute for Regeneration and Repair, The University of Edinburgh, Edinburgh, UK; 10 Cambridge Centre for Lung Infection, Royal Papworth Hospital and Department of Medicine, University of Cambridge, Cambridge, UK; 11 UCL Respiratory, University College London, London, UK; 12 Population Health Sciences Institute, Faculty of Medical Sciences, Newcastle University, Newcastle upon Tyne, UK

## Abstract

Bronchiectasis is characterised by permanent airway dilatation, airway infection, inflammation, chronic sputum production and repeated exacerbations [1]. Prevailing guidelines are largely empirical and note the critical lack of high-quality studies informing practice [2, 3]. Standard care is chest physiotherapy [1, 2, 4, 5] and antibiotics for treatment and prophylaxis of acute exacerbations [1, 2, 6].

## Shareable abstract

**This study comparing exacerbations rates for dual bronchodilator therapy, triple therapy and placebo in bronchiectasis cannot provide definitive evidence. However, results suggest signs of of efficacy: a larger trial may provide valuable clinical evidence.** https://bit.ly/4fuuHHX

*To the Editor:*

Bronchiectasis is characterised by permanent airway dilatation, airway infection, inflammation, chronic sputum production and repeated exacerbations [1]. Prevailing guidelines are largely empirical and note the critical lack of high-quality studies informing practice [2, 3]. Standard care is chest physiotherapy [1, 2, 4, 5] and antibiotics for treatment and prophylaxis of acute exacerbations [1, 2, 6].

Guidelines and systematic reviews highlight few large scale, adequately powered studies of inhaled therapies in bronchiectasis [1–3]. There is however widespread use of inhaled corticosteroids (ICS) and long acting bronchodilators such as long-acting beta agonists (LABA) and/or long-acting muscarinic antagonists (LAMA) [7].

ICS therapy is recommended in current guidelines for COPD in those with frequent exacerbations, especially when blood eosinophils are higher [8]; studies on COPD have shown a 25% reduction in exacerbations with ICS containing triple therapy (ICS/LAMA/LABA) *versus* LAMA/LABA dual bronchodilator therapy [8]. Triple therapy with fluticasone furoate, umeclidinium and vilanterol resulted in a lower rate of moderate or severe COPD exacerbations than fluticasone furoate–vilanterol (ICS/LABA) or umeclidinium–vilanterol (LAMA/LABA). Triple therapy in COPD also resulted in a lower rate of hospitalisation than LAMA/LABA [9]. COPD guidelines [8], however, highlight that ICS are not suitable for all patients with COPD and there is a higher likelihood of harms including increased rates of pneumonia, particularly in those with severe airflow limitation, prior history of pneumonia and low blood eosinophil status.

Combination bronchodilators and ICS have emerged in COPD [10] that may prove effective in bronchiectasis. The mechanism by which bronchodilators such as LAMA and LABA exert potentially beneficial effects in COPD is likely multifactorial. These have been recently reviewed and reach beyond reducing bronchoconstriction with effects on airway cilia, mucus production, airway nerve desensitisation and reducing gas trapping [11]. These effects mean long-acting bronchodilators may target mechanisms relevant to bronchiectasis and could offer benefit without the potential increased risk of pneumonia associated with ICS.

We undertook a pragmatic, multi-centre, placebo-controlled, three-arm, double blind, parallel group, prospective, randomised controlled trial to test the following: 1) dual therapy (LABA/LAMA) either as a stand-alone therapy or in combination with ICS (triple therapy: ICS/LABA/LAMA) is superior to placebo at reducing the number of protocol defined bronchiectasis exacerbations (per participant) requiring treatment with antibiotics during the 12 month treatment period; and 2) dual therapy is non-inferior to triple therapy at reducing the number of protocol defined bronchiectasis exacerbations (per participant) requiring treatment with antibiotics during the 12 month treatment period. The main study protocol was approved by a Health Research Authority Research Ethics Committee (reference: 21/ NE/0020) and has been published elsewhere [12]. Participants were recruited between 22 September 2021 and 21 October 2022 through respiratory departments at nine UK NHS (National Health Service) secondary care sites. We had a target sample size of 600 adults with bronchiectasis and a history of ≥2 exacerbations in any 12-month period within the preceding 24 months.

Participants were randomised to receive one puff daily for 12 months of either dual therapy (55 micrograms umeclidinium (LAMA) and 22 micrograms vilanterol (LABA)), triple therapy (as per dual therapy plus 92 micrograms fluticasone furoate (ICS)) or matched placebo dry powder inhalers in a 2:2:1 ratio.

The primary outcome was the number of participant reported bronchiectasis exacerbations requiring treatment with antibiotics during the 12 month treatment period (facilitated by a weekly diary). The primary economic outcome was incremental cost-per quality-adjusted life year gained at 12 months.

As part of a national review of studies around the COVID pandemic, the study was stopped early by the funder due to slow recruitment after 33 participants were randomised (14 dual therapy, 12 triple therapy and 7 placebo) – the study should have recruited 120 at this timepoint. The mean age was 69.4 (sd: 10.7) years with a range of 40–89. There was a slight female predominance (male to female ratio 45:55). Baseline disease severity showed severe bronchiectasis with a mean Bronchiectasis Severity Index (BSI) [13] score of 9.7 (sd: 4.6). 55% percent of participants (18 of 33) were classed as having severe bronchiectasis (BSI score of ≥9), with 45% (15 of 33) classed as having mild-moderate bronchiectasis (BSI score of 0–8). 11 participants had *Pseudomonas* colonisation at baseline (11 of 33 (33%)). The mean number of exacerbations in the preceding 12 month period was 3.2 (sd: 1.2). There was significant impairment of health-related quality of life with a mean St Georges Respiratory Questionnaire total score of 44.4 (sd: 21.0) and a Quality of life Bronchiectasis respiratory symptoms score of 57.1 (sd: 19.6). Notably, 14 of the 33 (42%) were ICS users at baseline (dual therapy: 6 of 14 (43%, triple therapy: 4 of 12 (33%), placebo: 4 of 7 (57%)). The median eosinophil count was 0.16 × 10^9·L^−1^ (interquartile range 0.10–0.27 (n=32)). Apart from sex and ICS use and some elements of medical history, baseline characteristics appeared approximately balanced across the groups. With small numbers, it is expected that some imbalances will exist, especially within binary variables where even one extra participant within a category can have a large impact on the percentage.

Of the 33 participants randomised, 30 (91%) completed follow-up at 12 months, three participants withdrew (dual therapy: 1 (7%); triple therapy: 1 (8%); placebo: 1 (14%)) and five participants discontinued therapy during the trial (dual therapy: 1 (7%); triple therapy: 2 (17%); placebo: 2 (29%)). Given the small sample size, the statistical and economic analyses are descriptive and exploratory. Exacerbation data were available for 32 (97%) participants (13 dual therapy, 12 triple therapy, 7 placebo). The median number of exacerbations during follow-up (the primary outcome) was 1 (interquartile range 0–3) for dual therapy, 2 (1–2.5) for triple therapy and 3 (2–3) for placebo (figure 1a) over a median of 12 months follow up (interquartile range 12.0–12.0; range: 2.5–12.7). The adjusted incidence rate ratio was 0.59 (95% CI 0.26–1.32) for dual therapy *versus* placebo and 0.54 (95% CI 0.23–1.27) for triple therapy *versus* placebo and 0.92 (95% CI 0.45–1.92) for triple therapy *versus* dual therapy (figure 1b). The lower quartile for time to first exacerbation was 88 days (95% CI 20–167) for dual therapy, 54 days (95% CI 13–81) for triple therapy and 13 days (95% CI 5–41) for placebo (figure 1c). Additionally, the median time to first exacerbation was 167 days for dual therapy compared to 81 days for triple therapy and 41 days for placebo. NHS resource use and quality-adjusted life year data were available on 30 (91%) of the randomised participants. There were no safety concerns identified.

**FIGURE 1:**
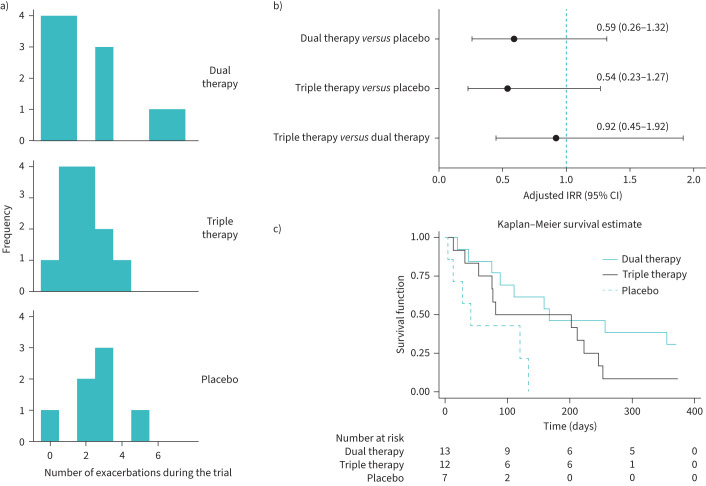
a) Histograms of number of bronchiectasis exacerbations requiring antibiotics during the trial per treatment group. Note that for the three participants who withdrew early, their number of episodes has not been adjusted for their shorter time in study. b) Comparison of the number of bronchiectasis exacerbations requiring antibiotics during the trial between treatment groups (n=32). The results are presented as incident rate ratios (IRRs) with a 95% confidence interval. These estimates are from fitting an exploratory negative binomial regression model adjusted for the stratification factors of Bronchiectasis Severity Index score and baseline inhaled corticosteroids drug therapy. The time at risk was considered to be the time not spent in exacerbation (hence, whilst a participant was experiencing an exacerbation they were not included as at risk of another). c) Kaplan–Meier estimates of survivor functions by treatment groups.

COVID-19 adversely impacted delivery of the trial by reducing staff capacity, limiting patient willingness to enter the study and by reducing exacerbation rates due to behavioural change in people with chronic respiratory disease. Additional challenges were a slower than planned site set-up and, ultimately, slow recruitment to the pilot. The trial is unable to provide evidence as intended on the superiority or cost-effectiveness of dual therapy or triple therapy to placebo at reducing mean exacerbation rates over 12 months, nor the non-inferiority of dual therapy to triple therapy in bronchiectasis. However, the results suggest there is a sign of efficacy. These results underscore the importance of completing a large-scale trial of these therapies to help improve the understanding of the best treatment for patients with bronchiectasis. This becomes more pressing as we understand more on the harms of injudicious use of ICS and better recognition of eosinophilic sub-population in bronchiectasis [14]. Some of the observed results may reflect the effects of ICS withdrawal with 42% of patients on ICS at baseline. Further analyses on this have not been conducted given the already small sample size. Future study designs should therefore strongly consider ICS-treated patients to be included within the eligible population and report outcomes based on this status. Although the sample size at termination was small there appeared to be more males recruited compared to many of the cohorts of bronchiectasis described. Future trials should consider carefully recruitment that represents patients seen in clinical practice and creates clear rules to avoid inadvertent recruitment of patients with COPD.

## Data Availability

The de-identified dataset from the trial will be prepared and stored by Newcastle University. Data sharing is subject to request providing detail of the purpose, analysis plan, results dissemination and authorship. If approved, data transfer is subject to completion of a Data Sharing Agreement between Newcastle University and the requester.
